# Gene expression profiling of breast cancer survivability by pooled cDNA microarray analysis using logistic regression, artificial neural networks and decision trees

**DOI:** 10.1186/1471-2105-14-100

**Published:** 2013-03-19

**Authors:** Hsiu-Ling Chou, Chung-Tay Yao, Sui-Lun Su, Chia-Yi Lee, Kuang-Yu Hu, Harn-Jing Terng, Yun-Wen Shih, Yu-Tien Chang, Yu-Fen Lu, Chi-Wen Chang, Mark L Wahlqvist, Thomas Wetter, Chi-Ming Chu

**Affiliations:** 1 Department of Nursing, Far Eastern Memorial Hospital & Oriental Institute of Technology, New Taipei, Taiwan; 2 Department of Surgery, Cathay General Hospital, Taipei, Taiwan; 3 Section of Biomedical informatics, School of Public Health, National Defense Medical Center, Taipei, Taiwan; 4 Department of Bioinformatics, Chung Hua University, Hsinchu, Taiwan; 5 Advpharma, Inc., Taipei, Taiwan; 6 School of Nursing, College of Medicine, Chang-Gung University, Taoyuan, Taiwan; 7 National Health Research Institute, Chunan, Taiwan; 8 Department of Medical Informatics, University of Heidelberg, Heidelberg, Germany

**Keywords:** Breast cancer, Microarray, Artificial neural network, Logistic regression, Decision tree

## Abstract

**Background:**

Microarray technology can acquire information about thousands of genes simultaneously. We analyzed published breast cancer microarray databases to predict five-year recurrence and compared the performance of three data mining algorithms of artificial neural networks (ANN), decision trees (DT) and logistic regression (LR) and two composite models of DT-ANN and DT-LR. The collection of microarray datasets from the Gene Expression Omnibus, four breast cancer datasets were pooled for predicting five-year breast cancer relapse. After data compilation, 757 subjects, 5 clinical variables and 13,452 genetic variables were aggregated. The bootstrap method, Mann–Whitney *U* test and 20-fold cross-validation were performed to investigate candidate genes with 100 most-significant p-values. The predictive powers of DT, LR and ANN models were assessed using accuracy and the area under ROC curve. The associated genes were evaluated using Cox regression.

**Results:**

The DT models exhibited the lowest predictive power and the poorest extrapolation when applied to the test samples. The ANN models displayed the best predictive power and showed the best extrapolation. The 21 most-associated genes, as determined by integration of each model, were analyzed using Cox regression with a 3.53-fold (95% CI: 2.24-5.58) increased risk of breast cancer five-year recurrence…

**Conclusions:**

The 21 selected genes can predict breast cancer recurrence. Among these genes, CCNB1, PLK1 and TOP2A are in the cell cycle G2/M DNA damage checkpoint pathway. Oncologists can offer the genetic information for patients when understanding the gene expression profiles on breast cancer recurrence.

## Background

Breast cancer is one of the most common cancers in women worldwide. According to the American Cancer Society, breast cancer is the second leading cause of death among women in the U.S.[1]. However, significantly different five-year recurrence rates and survival rates have been observed among breast cancer patients with the same course of disease. In other words, prognostic factors for breast cancer recurrence, such as histology and lymph node status, cannot entirely correctly predict the subsequent clinical manifestations of patients [2,3].

Microarray technology can be used to acquire information about thousands of genes simultaneously. Traditional statistical methods, such as logistic regression, have become increasingly difficult to use for survivability prediction models due to several constraints that dictate the low statistical power with small sample size and complex polynomial interaction terms with curvilinear effects among the relationship of variables. Data mining techniques, such as artificial neural networks and decision trees, can process thousands of independent variables without the need to consider constraints from statistical assumption and polynomial interaction terms. Compared with logistic regression, these techniques have a better potential and are more advantageous for building survivability prediction models.

Previously reported analyses of microarray data that aimed to predict breast cancer recurrence rarely selected the same groups of genes, possibly due to the small sample sizes used [4-11]. One of the objectives of the present study was to increase the sample size through the integration of samples from multiple breast cancer microarray databases. In addition, we sought to assess the capacity of logistic regression, decision tree and artificial neural network models to predict breast cancer recurrence, with the goal of developing a more predictive gene profile for breast cancer relapse within five years and identifying important risk genes that affect breast cancer recurrence.

## Methods

### Data sources

**Figure 1:**
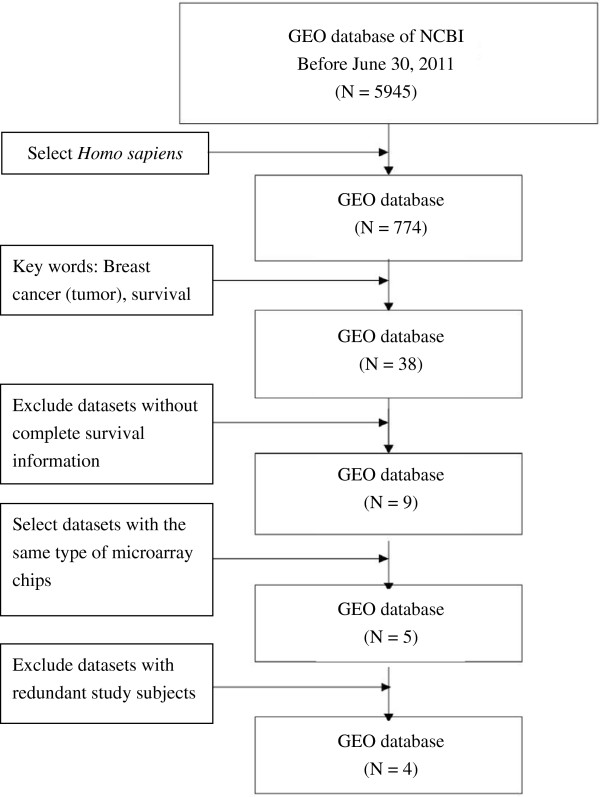
Flow chart showing the protocol used for the search and download of breast cancer microarray datasets from the GEO database.

The present study used microarray datasets from the Gene Expression Omnibus (GEO) database of the National Center for Biotechnology Information (NCBI) of the U.S. National Library of Medicine. The search process for the datasets included in the study is shown in Figure 1. There were 5,945 datasets in the microarray database provided by GEO before June 30, 2011, of which 774 datasets were derived from *Homo sapiens*. A total of 38 datasets were obtained when “breast cancer (tumor)” and “survival” were used as keywords to search the 774 datasets. After excluding 25 datasets without clinical survival information and four datasets without clinical variable codebook or survival time variables, nine datasets were left. Of these nine remaining datasets, five used the same type of microarray chip (HG-U133A). Of these five breast cancer microarray datasets, four [11-14] were ultimately included in the present study (Table 1); the remaining dataset was excluded because its study subjects overlapped with those of the other datasets.

**Table 1:** Breast cancer microarray datasets

**GEO number**	**Year**	**Author**	**Paper title**	**Chip type**	**Number of study subjects**
GSE7390	2007	Desmedt et al. [12]	Strong time dependence of the 76-gene prognostic signature for node-negative breast cancer patients in the TRANSBIG multicenter independent validation series.	HG-U133A	198
GSE2990	2006	Sotiriou et al. [14]	Gene expression profiling in breast cancer: understanding the molecular basis of histologic grade to improve prognosis.	HG-U133A	189
GSE4922	2006	Ivshina et al. [13]	Genetic reclassification of histologic grade delineates new clinical subtypes of breast cancer.	HG-U133A	249
GSE2034	2005	Wang et al. [15]	Gene-expression profiles to predict distant metastasis of lymph-node-negative primary breast cancer.	HG-U133A	286

Abbreviations: GEO, Gene Expression Omnibus; GSE, GEO Datasets Number Prefixes; HG-U133A, a type of oligonucleotide Gene Chip from the Affymetrix.

### Preprocessing of microarray data

The four breast cancer microarray datasets included in this study all employed the HG-U133A oligonucleotide Gene Chip from Affymetrix. This array is comprised of 22,283 probes for the simultaneous analysis of 20,000-30,000 genes. In our meta-analysis, the probe data of the four datasets were analyzed to obtain log conversions, standardized Z values, the sum of each probe score and quartile rankings for subsequent study. We used the GC Robust Multi-array Average (GCRMA) method and R language software with procedures of library(gcrma) and library(precprocessCore) to remove the chip background associated with the microarray gene expression levels. The expression levels of the probe sets were converted into gene expression levels. Because the probe expression levels showed a skewed distribution, the median probe expression in a gene was calculated to represent the gene expression level. The datasets were finally merged to obtain the expression levels of genes, which conversion formulae 1–3 followed by the quantile normalization [16,17] of all gene expression values.

The Desmedt [12] dataset was selected as the reference standard in this study. The other three datasets [13-15] (the Wang et al., Sotiriou et al. and Ivshina et al. datasets) were subjected to log conversion so that they were similar to the Desmedt et al. [12] dataset in terms of central tendency (mean), dispersion tendency (maximum, minimum, range and standard deviation), skewness and kurtosis. The conversion formulae for the chip value of each dataset were as follows:

(1) Sotiriou et al. [14]

(Formula1) $$Y={\mathrm{Log}}_{2}\left(2.2^{X}-8\right)$$

(2) Ivshina et al. [13]

(Formula2) $$Y={\mathrm{Log}}_{2}\left(2.65^{X}-0.1\right)$$

(3) Wang et al. [15]

(Formula3) $$Y={\mathrm{Log}}_{2}\left(X\right)$$

X: original value, Y: converted value.

Following log conversion, the four datasets were further standardized into Z values with a mean value of 0 and a standard deviation of 1. Compared with the original data, the standardized Z values did not show significant differences in distribution among study objects.

The HG-U133A gene chip used in this study is comprised of 22,283 probes that cover 13,452 genes. Each gene is covered by 1–14 probes. Of the 13,452 genes, 5,107 (38%) are covered by more than two probe combinations. For genes covered by multiple probe combinations, this study adopted the median method. For example, when the expression level of the HFE gene was reflected by the levels of 13 probes, the level of the seventh (the median number) probe was chosen to represent the expression of the HFE gene.

### Definition and selection of clinical variables

The clinical and survival variables provided by the four datasets of this study are shown in Table 2. The dependent variable is defined as death from breast cancer within five years or any form of relapse, as evidenced by, for example, local lymphatic drainage or distant metastases.

**Table 2:** Clinical variables of each dataset

**Item**	**Wang et al. [**[15]**]**	**Ivshina et al. [**[13]**]**	**Sotiriou et al. [**[14]**]**	**Desmedt et al. [**[12]**]**	**Combined database**
Clinical data	Lymph node status	Lymph node status	Lymph node status	Lymph node status	Lymph node status
	Estrogen receptor	Estrogen receptor	Estrogen receptor	Estrogen receptor	Estrogen receptor
		Age	Age	Age	Age
		Tumor size	Tumor size	Tumor size	Tumor size
		Histopathologic grade	Histopathologic grade	Histopathologic grade	Histopathologic grade
Treatment	Estrogen therapy	Estrogen therapy	Tamoxifen therapy	Surgical therapy	Surgical therapy
	Adjuvant therapy	Adjuvant therapy	Surgical therapy		Tamoxifen therapy
	Surgical therapy	Surgical therapy			Estrogen therapy
					Adjuvant therapy
Survival	Distant metastasis events	Relapse events^a^	Relapse events ^b^	Relapse events ^b^	Relapse events ^a^
	Distant metastasis time	Relapse time	Relapse time	Relapse time	Relapse time
			Distant metastasis events	Survival events	
			Distant metastasis time	Survival time	
				Distant metastasis events	
				Distant metastasis time	

^a^ Data includes death by breast cancer or any form of breast cancer recurrence (including local lymphatic drainage and distant metastases).

^b^ Data includes any form of breast cancer recurrence (including local lymphatic drainage and distant metastases).

### Study subject selection

**Figure 2:**
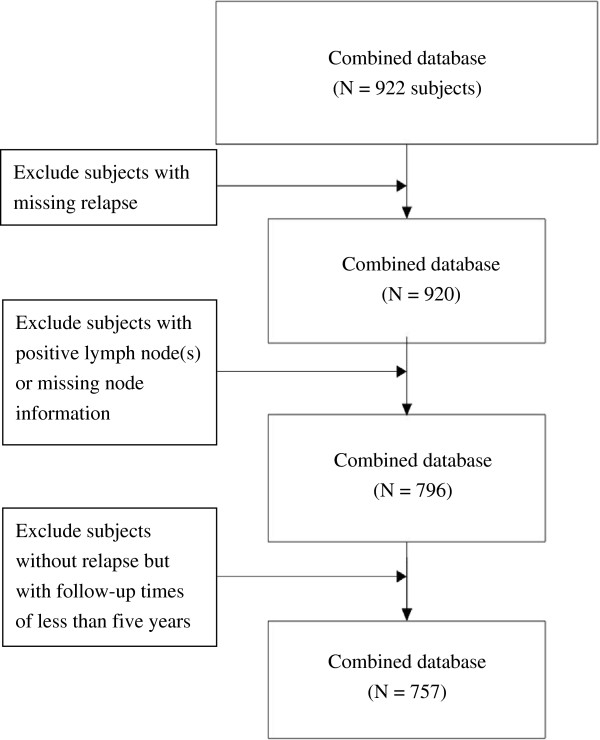
Flow chart of the protocol used for study subject selection.

The merged dataset of this study consisted of 922 study subjects. Two of the subjects were excluded due to the missing value of relapse status (dependent variable). Within the merged dataset, the Sotiriou et al. dataset had 34 cases with positive lymph nodes or missing values, and the Ivshina et al. dataset had 90 cases with positive lymph nodes or missing values. Positive lymph nodes are an important factor that affects breast cancer relapse and survival. Lymph node-negative patients are clinically classified as early-stage patients, with better survival rates and recurrence rates than lymph node-positive patients. The present study intended to identify the risk genes that can be used to effectively predict the future risk of breast cancer relapse at the early stage of breast cancer pathogenesis. Therefore, the 124 study subjects with positive lymph nodes or missing values were excluded from this study, and 796 patients were included. In addition, previous studies indicated that 75% of breast cancer patients experienced relapse within the first five years. To avoid insufficient follow-up time, 39 subjects with follow-up times shorter than five years were excluded; thus, a total of 757 subjects were included in the present study (Figure 2).

### Gene prediction model

**Figure 3:**
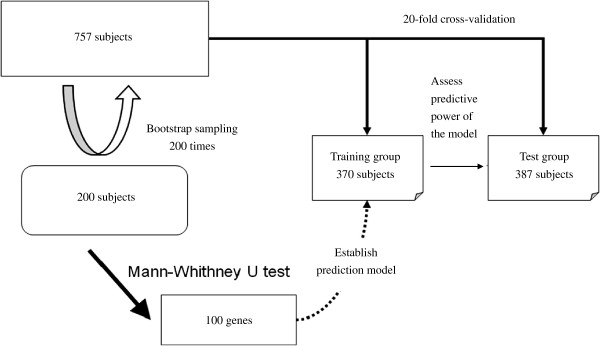
Diagram of the methods used to identify predictive genes and establish prediction models.

A total of 200 subjects were randomly selected from the combined dataset of this study for the selection of gene loci using the Mann–Whitney *U* test. For the test, 200 rounds of bootstrapping were performed. For each gene, the top and bottom 5% of P values were eliminated, and a mean was calculated for the left 180 P values. The obtained mean P values were ranked in order. The first 100 genes with the smallest P values were included in the decision tree, artificial neural network and logistic regression models (Figure 3). The 757 study subjects from the combined dataset were randomly assigned as the training samples or the test samples for 400 rounds, with 370 training samples and 387 test samples in each dataset.

The 100 genetic variables and six clinical variables (age, tumor size, histopathological classification, estrogen receptor status, relapse occurrence within five years and relapse onset time) from the 400 training and test sample sets were subjected to statistical tests. The variables without significant differences between the training and test samples were selected to establish 20 training sets for a 20-fold cross-validation.

### Construction of the prediction models

In this study, Clementine 10.1 was used to construct the decision tree (DT), logistic regression (LR) and artificial neural network (ANN) models. The ANN parameter of over-training prevention was set as the percent difference between the training samples and test samples. The prediction accuracy of the test group was higher than that of the training group without the setup of the over-training prevention parameter. Therefore, the ANN model of this study was set at 80% over-training prevention, or ANN80. Because LR does not have the option of over-training prevention, another ANN model, ANN100, was constructed without over-training prevention to compare the predictive power of LR and ANN.

Because DT is capable of selecting important variables from a field of many, the composite model of this study first used DT to select important variables, which were then integrated into the LR or ANN models. Three types of composite models were used: the DT-LR composite model (DL), the DT-ANN composite model with 80% over-training prevention (DA80) and the DT-ANN composite model without over-training prevention (DA100).

### Criteria for assessing the results of the analysis

Three indicators were adopted to evaluate the predictive ability of the models in this study. The first indicator was accuracy (ACC). For this measure, the higher the score, the better the predictive ability of the model. ACC was calculated as follows:

ACC = number of cases with correctly predicted breast cancer recurrence within five years/total number of cases.

The second indicator was AUC: the area under the ROC curve drawn by sensitivity (X axis) vs. 1-specificity (Y axis). This value can be used to determine the classification ability of a model: the higher the AUC, the better the predictive ability of the model.

The third indicator was extrapolation, or the difference in ACC (or AUC) between the training and test samples. This value represents the magnitude of change in the predictive ability of a prediction model toward test samples after training with the training samples. This value was calculated as follows:

ΔACC = ACC of training samples – ACC of test samples

ΔAUC = AUC of training samples – AUC of test samples

### Analysis of recurrence risks and genetic and biochemical pathways

In this study, SPSS14.0 software was used to perform a Cox proportional regression to analyze the relative risks of genetic characteristics with regard to breast cancer recurrence. The log-rank test was used to determine the survival curve variances of genetic characteristics, and the Ingenuity Pathway Analysis database was used to analyze and predict the major biochemical functions of the identified genes. The net reclassification improvement (NRI), which is available in a sub function of the MATLAB [18], was used to compare AUC for cox models that contained the predictors and those that did not, as additional markers of incremental improvement in risk prediction [19-21].

## Results

In this study, we determined the average predictive power of each model by using a 20-fold cross-validation. For the training samples, DT showed the highest predictive power; ANN100 and LR had similar predictive powers, and the accuracy of ANN80 was the lowest. In terms of the predictive power for test samples and extrapolation, ANN80 was the best and DT was the worst (Table 3). Among the composite models, DA100 displayed the best predictive power for the training samples, while DA80 showed the lowest accuracy. With regard to predictive power and extrapolation within the test samples, DA80 was the best model, while DA100 was the worst model (Table 4).

**Table 3:** Assessment of the predictive power of each single model using the 100-gene profile

**Prediction model**	**ACC**			**AUC**		
	**Training sample**	**Test sample**	^**Δ **^**(Difference)**	**Training sample**	**Test sample**	^**Δ **^**(Difference )**
DT	93.63	63.45	30.18	94.02	56.90	37.13
LR	82.53	64.12	18.40	87.68	58.96	28.72
ANN80	73.42	70.93	4.09	72.11	64.09	8.02
ANN100	84.63	69.54	15.09	84.98	63.88	21.09

Abbreviations: ACC, ACCuracy; AUC, Area Under the Curve; DT, Decision Tree; LR, Logistic Regression; ANN80, Artificial Neural Network using 80% resampling set (over-training prevention); ANN100, ANN using 100% resampling set (without over-training prevention).

**Table 4:** assessment of the predictive power of the composite models using the 100-gene profile

**Prediction model**	**ACC**			**AUC**		
	**Training sample**	**Test sample**	^**Δ **^**(Difference)**	**Training sample**	**Test sample**	^**Δ **^**(Difference)**
DL	75.60	68.90	6.69	77.59	61.66	15.93
DA80	72.69	69.30	3.39	71.92	64.20	7.72
DA100	89.91	65.91	22.56	87.74	61.65	26.10

Abbreviations: ACC, ACCuracy; AUC, Area Under the Curve; DL, Decision Tree combined with Logistic regression; DA80, Decision tree combined with Artificial neural network using 80% resampling set (over-training prevention); DA100, Decision tree combined with Artificial neural network using 100% resampling set (without over-training prevention).

The top 10 most important genes based on rankings from all of the single models tested in this study were as follows: LMCD1, DEAF1, AP2A2, LMNB1, ZFP36L2, ABCC1, PLOD2, LARS2, CDCA3 and AACS. Of these, LMCD1 was ranked first in three of the four models (LR, ANN80 and ANN100), DEAF1 was ranked among the top 10 most important genes in all models and AP2A2 was ranked among the top 10 most important genes in three models (DT, ANN80 and ANN100). The overall top 10 important genes in all models were among the top 40% of important genes in any single model.

**Figure 4:**
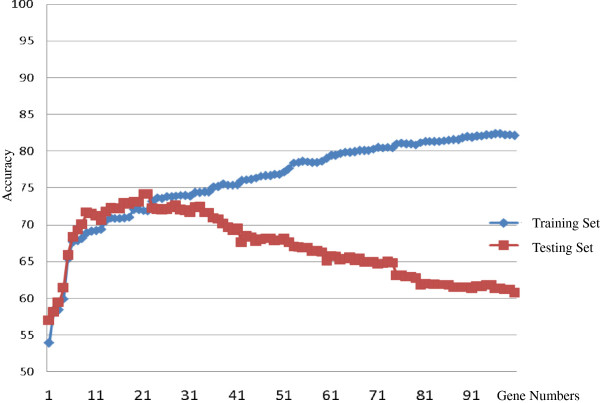
The AUC values of different gene numbers and the Cox regression of five-year recurrence rates of the test samples.

The relative importance of the genes in each model was integrated, and a group of samples in which there was no significant difference among the 100 genes was re-selected as the training set. Next, the genes were incorporated into the training sample group in order of importance for a Cox proportional hazards model, and risk scores for five-year breast cancer recurrence of the training and test samples were calculated via standardized Cox regression coefficients obtained from the training samples. The correlation of the obtained recurrence scores with the predictive power for five-year breast cancer recurrence is shown in Figure 4. The predictive ability AUC of the training samples gradually increased with an increase of the number of genes, while the prediction ability of the test samples displayed quadratic characteristics. At first, the predictive ability increased as the number of genes increased, but it then started to decrease when the number of genes reached a certain value. As shown in the Tables 3 and 4, the AUC in the test dataset ranged from 0.61 to 0.64 using the 100 genes, it implicates that would be consistent with marginal predictive ability at best and should refrain from recommending such a model for use in clinical practice. Therefore, the present study found and proposed that 21 genes had the highest predictive power among the test samples, with an AUC value of 0.7412 in Figure 4.

**Figure 5:**
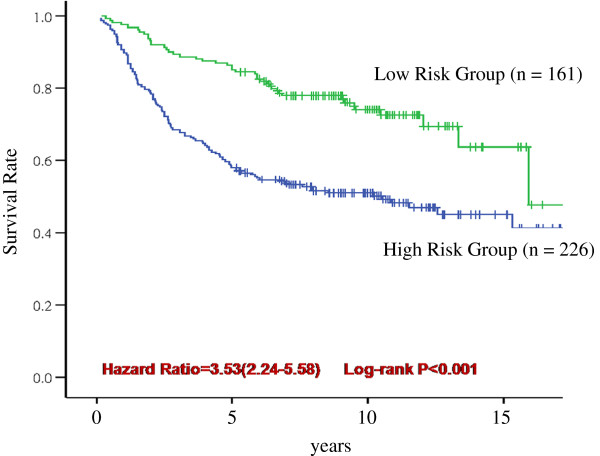
Kaplan-Meier analysis of 21 gene expression profile.

In this study, the sensitivity of the training samples for five-year breast cancer recurrence was set above 80%, and the recurrence score of the 21 genes at the highest specificity was set as the cutoff point. The study subjects were divided into high- and low-risk recurrence groups, and Cox regression was used to analyze the 21 genes for the prediction of breast cancer recurrence and survival rate in each group (Figure 5). The results showed a statistically significant difference (P < 0.001) in the recurrence time curve between the two groups.

The breast cancer recurrence risk (HR value) of the high-risk group was 3.53 times that of the low-risk group. We used test samples to assess the risk of breast cancer recurrence via the 21 predictive genes and clinical variables. As shown in Table 5, after adjusting for other clinical variables through multivariate Cox regression, a statistically significant HR value for the prediction of five-year breast cancer recurrence risk using the 21 identified genes was 2.60 (95% CI = 1.44-4.68). Pencina et. al, proposed NRI has been proven as an alternative to the area under the curve of the ROC [19-21]. In the multivariate Cox regression, the 21-gene profile has a 0.454 NDI greater than age, tumor diameter, histopathologic grade and estrogen receptor. It concludes that addition of the 21-gene profile improved classification for a net of 45.4 per cent of the five-year breast cancer recurrence.

**Table 5:** Cox regression analysis of the five-year breast cancer recurrence of the test samples

**Item**	**Univariate**		**Multivariate**		
	**HR (95% CI)**	**P**	**HR (95% CI)**	**P**	**NDI**
21 Genes Profile	3.53 (2.24-5.58)	<.001	2.60 ( 1.44-4.68)	.001	0.454
Age	0.98 (0.96-1.00)	.115	0.99 (0.977-1.02)	.896	0.012
Tumor Diameter	1.54 (1.21-1.95)	<.001	1.41 (1.07-1.86)	.013	0.121
Histopathologic grade^a^	4.85 (1.94-12.09)	.001	3.59 (1.41-9.16)	.007	0.182
Estrogen Receptor ^b^	1.50 (1.02-2.20)	.035	1.03 (0.58-1.82)	.902	0.035

Abbreviations: HR, Hazard Ratio. CI, Confidence Interval. P, statistical P value. NDI, Net Reclassification Improvement. a, Histopathologic grade: assessed by the Nottingham grading system. b, Estrogen receptor: negative or positive.

**Figure 6:**
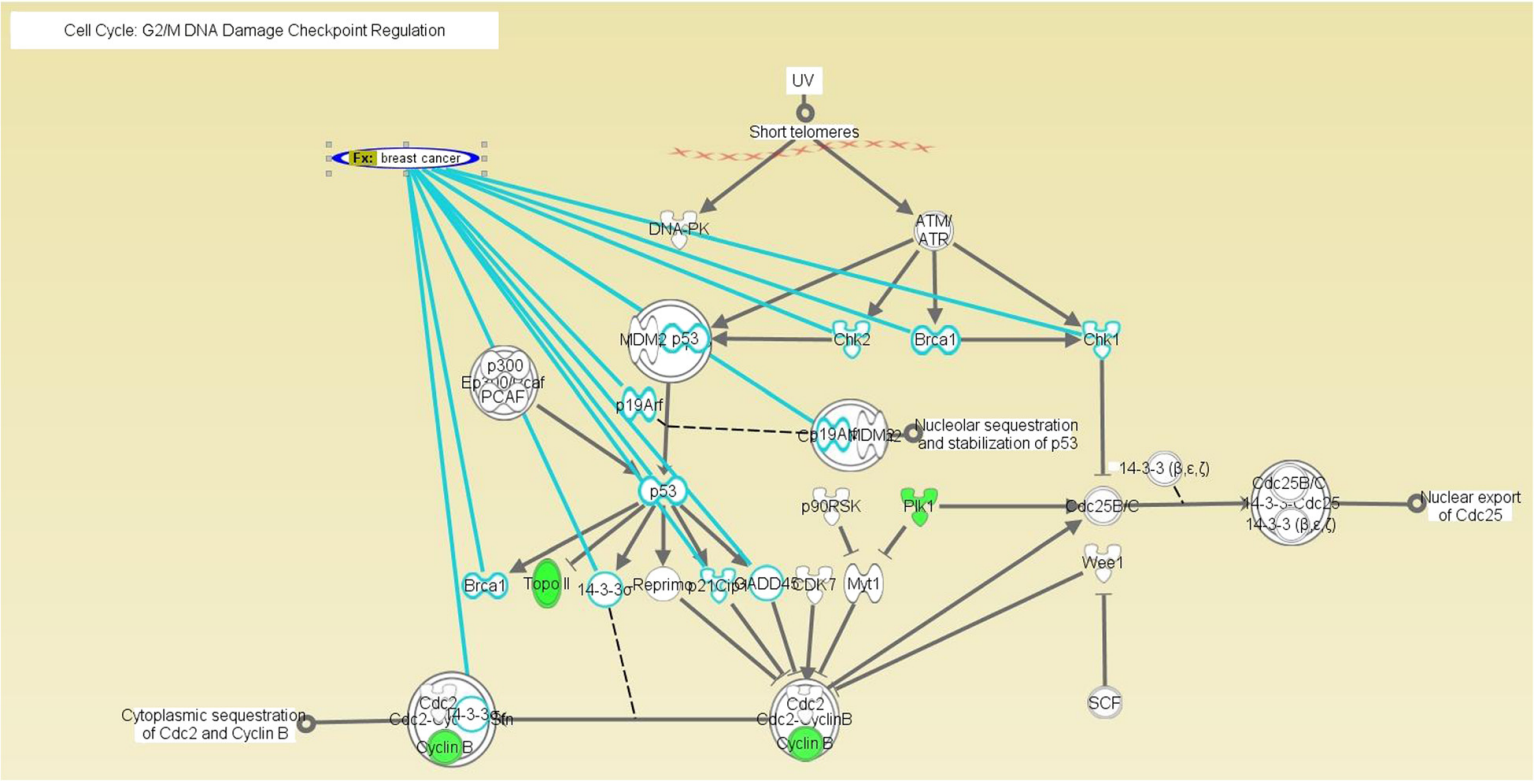
**Breast cancer-related genes and DNA damage checkpoint regulation at the G2/M phase of the cell cycle.** ANN: Artificial Neural Network; DA: Decision Tree combined with ANN; LR: Logistic Regression; DL: Decision Tree combined with LR; DT: Decision Tree.

The Ingenuity Pathway Analysis database was used to analyze the biochemical pathways with which the 100 identified genes are associated. Of these 100 genes, 28 are related to pathways involved with cancer, the cell cycle and reproductive system diseases; 15 are related to pathways involved in cell morphology, cellular compromise and cancer; 15 are related to cell cycle, amino acid metabolism and post-translational modification pathways; and 14 are related to cell cycle, cellular movement and cancer pathways. A total of 65 genes found in this study function in biochemical pathways related to the cell cycle and cancer; of these, 28 genes (the largest group and those with the highest scores) are involved in pathways related to cancer, the cell cycle and reproductive system diseases. Therefore, these 100 breast cancer recurrence-related genes most likely participate in pathways that regulate the DNA damage checkpoint at the G2/M phase of the cell cycle. Figure 6 shows the relationships of the 11 reported breast cancer-related genes and proteins with the regulation of the DNA damage checkpoint at the G2/M phase of the cell cycle. Three genes identified in this study, CCNB1, PLK1 and TOP2A (shown in green), were also found to be involved in this biochemical pathway. This finding suggests that the genes identified in this study are associated with the same biochemical pathways as the genes involved in breast cancer and thus may have impacts on breast cancer development.

## Discussion

**Figure 7:**
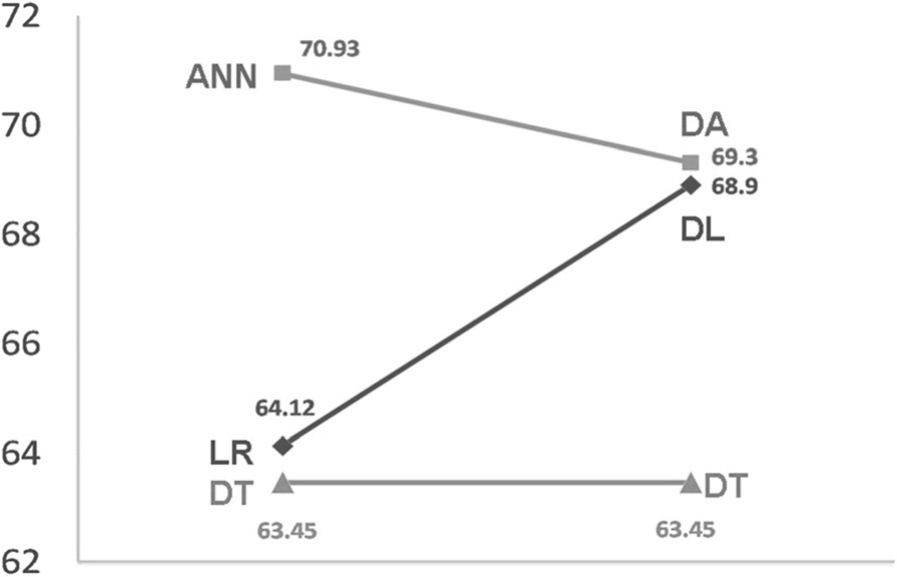
Accuracy ratio between single and composite models.

In previous studies, Delen et al. and Snow et al. compared artificial neural network models and logistic regression in terms of their abilities to predict five-year cancer survival rates. These authors found that the AUC results of internal validation (using training samples) for the artificial neural network model were superior to those of logistic regression, a finding that is consistent with the results of the present study [16,22-25]. Xu et al. found that composite models with decision trees can effectively screen the characteristics of variables and that the input of the important variables selected in a decision tree model into other models can improve the extrapolation of the model. If the predictive power of a decision tree is far worse than that of the other model, the prediction accuracy of the composite model will not be improved, but its extrapolation will still be improved [26]. In the present study, the accuracy of the test samples was used to assess the predictive power of the single and composite models. As shown in Figure 7, when logistic regression had a similar predictive power to that of the decision tree, the predictive power of the logistic composite model increased. However, when the predictive ability of the decision tree was much lower than that of the artificial neural network, the predictive ability of the composite model decreased.

When we investigated the reproducibility of the selected genes from each dataset, we found that only three genes, CCNE2, GTSE1 and KPNA2, were included in the lists of genes selected by the original authors, suggesting a very low reproducibility of the selected genes in different studies. The 100 genes selected in this study were compared with the genes selected by the original authors. The results showed that 5 genes from this study were also selected by Wang et al. and Desmedt et al., 19 genes were also selected by Sotiriou et al. and 20 genes were also selected by Ivshina et al.. Among these genes, two genes, MLF1IP and PLK1, were selected by Wang et al., Desmedt et al., Sotiriou et al. and this study. The PLK1 gene was ranked among the top 10 most important genes in the DT and ANN100 models.

Xu et al. analyzed genes related to five-year metastasis rates of breast cancer using four breast cancer microarray datasets that are available online [27]. Of these four datasets, two (Wang et al. and Sotiriou et al.) were also included in the present study. In addition, the Miller et al. dataset is essentially equivalent to the Ivshina et al. dataset included in this study because only three subjects differed between the two datasets. Thus, the Desmedt et al. dataset is the only dataset that was included in the present study but not included in the 2008 study by Xu et al. (the Pawitan et al. dataset [28] was the fourth dataset used in the Xu et al. study). Of the 112 predictive genes identified by the Xu et al. study, 5 genes were also selected by Wang et al. and Desmedt et al., 19 genes were also selected by Sotiriou and 19 genes were also selected by Ivshina; this level of agreement is similar to that observed in the present study. In this study, we compared our 100 selected genes with the 112 genes from the 2008 Xu et al. study. We found that 13 genes were selected in both studies: AP2A2, ASPM, CDKN3, EEF1E1, IGHM, IGKC, LST1, MAD2L1, MELK, MLF1IP, PRC1, RACGAP1 and STK6. Xu et al. addressed the same question as the present study and conducted a meta-analysis using similar databases. The percentage of identified genes that overlapped with those in the original datasets is similar between our study and the Xu et al. study, suggesting that, when compared with genes selected using small sample sizes, selecting genes by meta-analysis can improve the accuracy of predicting breast cancer recurrence.

Xu et al. predicted five-year breast cancer recurrence rates using 112 selected genes, achieving a sensitivity of 88% and a specificity of 54.6%. The risk of recurrence was 9.3-fold (hazard ratio = 9.3, 95% CI: 2.9-29.9). The risk of five-year breast cancer recurrence found in this study was lower than that found in the 2008 study by Xu et al. However, this study achieved a similar result to that study with only one-fifth the number of genes, suggesting that the 21 genes in this study can effectively differentiate breast cancer patients with high and low risks of recurrence.

The present study observes a proportion of genes consistently identified by pooled microarray datasets of aggregating several studies that can be a set of candidate genes expression profile for a future work. It is very important to examine how reliable the set of signatures proposed in this study can predict cancer relapse of breast cancer patients in an independent replication study. Furthermore, the candidate genes also are worthy to investigate more characteristics on epigenetics and genetics in breast cancers, like as DNA methylations, mRNA expressions, micro RNA interactions, biochemical pathway and so on, for future studies.

One limitation of this study is that the pooled microarray datasets were obtained from multiple studies. It can benefit from an increase of sample size but may also compensate for study heterogeneity caused by the discrepancies among studies. They lacked the complete collection to identify the discrepancies of all breast cancer-related variables among studies, as well as variables affecting the survival of breast cancer patients, such as the use of chemotherapy and radiotherapy, phenotype definition, population ethnicity, genetic heterogeneity. Therefore, this study did not effectively control for other breast cancer-related factors that could affect the selection of the genes related to breast cancer recurrence. However, we tried to adjust causes by the discrepancies among studies, like as age, tumor diameter, histopathologic grade and estrogen receptor. Although four datasets were combined to increase the sample number in this study, only 757 patients were left after excluding those patients with positive lymph nodes or follow-up times of less than 5 years. Additionally, several other groups of study subjects, such as those treated with tamoxifen, chemotherapy or radiotherapy and those with redundant database entries, were not excluded to ensure an adequate number of samples for the study. The authors of the original datasets mentioned that the inclusion of patients who received these treatments would not affect the results of their studies; thus, in the present study, we assumed that the breast cancer recurrence rates and gene expression levels among the selected patients were not affected by interfering factors, in the absence of more detailed information.

## Conclusion

In the present study, after integrating the results of breast cancer microarray dataset analyses using several different models, we identified 21 genes that are closely related to breast cancer recurrence: LMCD1, DEAF1, AP2A2, LMNB1, ZFP36L2, ABCC1, PLOD2, LARS2, CDCA3, AACS, TNFRSF25, SMC1A, ADIPOQ, DPP3, FADD, PLK1, SDS, HSPB6, MTERFD1, CHPF and AQP1. Among these, the PLK1 gene was of particular interest because it is involved in the DNA damage checkpoint response at the G2/M phase of the cell cycle and, along with other genes (such as CCNB1 and TOP2A), plays a role in regulating cell cycle progression. Regarding statement of translational relevance, we concluded the most effective genes profiling and identified 21 genes that are closely related to breast cancer recurrence: LMCD1, DEAF1, AP2A2, LMNB1, ZFP36L2, ABCC1, PLOD2, LARS2, CDCA3, AACS, TNFRSF25, SMC1A, ADIPOQ, DPP3, FADD, PLK1, SDS, HSPB6, MTERFD1, CHPF and AQP1. Among these, the PLK1 gene was of particular interest because it is involved in the DNA damage checkpoint response at the G2/M phase of the cell cycle and, along with other genes (such as CCNB1 and TOP2A), plays a role in regulating cell cycle progression. Two genes, MLF1IP and PLK1, were selected by the most pooled microarray datasets of Wang et al., Desmedt et al., Sotiriou et al. and this study. The PLK1 gene was ranked among the top most reproductive gene. These genes profiling will be valuable to be as the targets of prognosis and treatment.

## Abbreviations

ACC: Accuracy; AUC: Area under the curve; DT: Decision tree; LR: Logistic regression; ANN: Artificial neural network; GEO: Gene Expression Omnibus

## Competing interests

The authors declare that they have no competing interests.

## Authors’ contributions

HLC, CTY, CYL and CMC implemented the method and wrote the manuscript. HLC, KYH, HJT and CMC designed the experiments, evaluated the experimental results, and assessed their statistical significance. HLC, CYL, KYH and CMC generated and preprocessed the biological data, and also interpreted the resulting analysis. MLW, TW and CMC interpreted and validated the findings. All authors read and approved the final manuscript.
